# Prognostic value of HLA class I expression in patients with colorectal cancer

**DOI:** 10.1186/s12957-015-0456-2

**Published:** 2015-02-12

**Authors:** Yuji Iwayama, Tetsuhiro Tsuruma, Toru Mizuguchi, Tomohisa Furuhata, Nobuhiko Toyota, Masayuki Matsumura, Toshihiko Torigoe, Noriyuki Sato, Koichi Hirata

**Affiliations:** Department of Surgery, School of Medicine, Sapporo Medical University, S1, W16, Chuo-ku, Sapporo, Hokkaido 060-0061 Japan; Department of Pathology, School of Medicine, Sapporo Medical University, S1, W16, Chuo-ku, Sapporo, Hokkaido 060-061 Japan

**Keywords:** HLA class I, Colorectal cancer, Prognostic factor, Relapse, Disease-free survival

## Abstract

**Background:**

Prognostic factors are useful for determination of the therapeutic strategy and follow-up examination after curative operation in cancer treatment. The immunological state of the host can influence the prognosis for cancer patients as well as the features of the cancer. Human lymphocyte antigen (HLA) class I molecules have a central role in the anti-cancer immune system. Therefore, we focused on the HLA class I expression level in cancer cells to investigate its prognostic value in patients with colorectal cancer.

**Methods:**

We reviewed the clinical pathology archives of 97 consecutive patients with stage II colorectal cancer who underwent curative operation at the Sapporo Medical University, Japan, from February 1994 to January 2005. Fifty-six high-risk patients had adjuvant chemotherapy. The cancer cell membrane immunoreactivity level for HLA class I expressed by EMR8-5 was classified into three categories (positive, dull, and negative). In this study, the cases were divided into two groups: “positive” and “dull/negative”. HLA class I expression level and clinicopathological parameters were evaluated with the Pearson *χ*^2^ test. Survival analysis was assessed by the Kaplan-Meier methods, and the differences between survival curves were analyzed using the log-rank test.

**Results:**

Immunohistochemical study of HLA class I revealed the following. There were 51 cases that were positive, 40 were dull, and six negative. The HLA class I expression level had no significant correlation with other clinicopathological parameters, except for gender. Univariate and multivariate analyses related to disease-free survival (DFS) revealed that tumor location, HLA expression level, and venous invasion were significant independent prognostic factors (*P* < 0.05). The 5-year DFS rates in HLA class I positive group and in the dull/negative group were 89% and 70%, respectively. For high-risk patients with adjuvant chemotherapy, the 5-year DFS rates in the HLA class I positive group and in the dull/negative group were 84% and 68%, respectively. For low-risk patients without the chemotherapy, the 5-year DFS rates in the HLA class I positive group and in the dull/negative group were 100% and 71%, respectively.

**Conclusions:**

Our study concluded that the HLA class I expression level might be a very sensitive prognostic factor in colorectal cancer patients with stage II disease.

## Background

Recently, the number of patients with colorectal cancer is increasing. Colorectal cancer is the third most common cancer and the fourth most frequent cause of cancer death worldwide [1]. In Japan, colorectal cancer is the third leading cause of cancer-related death.

The prognosis is related to many histopathological and clinical parameters, with the most important prognostic factor affecting survival for patients undergoing curative operation being the presence or absence of regional lymph node involvement [2]. Therefore, it is generally recommended that patients with stage III colorectal cancer, which includes regional lymph node metastases, should undergo adjuvant chemotherapy. However, controversy still exists regarding the necessity of adjuvant chemotherapy for node-negative patients with stage II disease [3]. The QUASAR trial demonstrated that adjuvant chemotherapy with fluorouracil/leucovorin (FU/LV) could improve survival of patients with stage II colorectal cancer, although the absolute improvements were small [4]. Pooled analysis (IMPACT B2) of randomized trials comparing groups with adjuvant chemotherapy receiving FU/LV and those with surgery alone demonstrated that there was no significant difference in event-free and overall survival [5]. Meanwhile, O’Connor et al. reported that no 5-year survival benefit from adjuvant chemotherapy was observed for patients with stage II disease, although a benefit was observed for those with stage III disease [6]. In the present situation, adjuvant chemotherapy is conducted for patients categorized into a high-risk group among those with stage II disease on the basis of various histopathological or clinical parameters such as poorly differentiated histology, lymphovascular invasion, perineural invasion, T4 tumor stage, bowel obstruction or perforation, and an elevated preoperative plasma level of carcinoembryonic antigen (CEA) [7]. These parameters are indicated in some guidelines such as the National Comprehensive Cancer Network (NCCN), European Society for Medical Oncology (ESMO), etc. [8], although they are not based on conclusive evidence.

The immune system discriminates between self and nonself, targeting, for example, cancer cells. However, cancer cells can escape from the immune system and grow, metastasize, and finally cause death. One mechanism of the immune escape by cancer development is the downregulation of human lymphocyte antigen (HLA) class I molecules, which are cancer antigen-presenting molecules for cytotoxic T lymphocytes (CTLs) [9-12]. The immune state is of great importance in the prognosis of cancer patients. Therefore, we focused on the HLA class I expression level in cancer cells to investigate its prognostic value in patients with colorectal cancer. Since most anti-HLA class I antibodies recognize the allele-specific native structure of HLA class I molecules, these antibodies have been unable to react with denatured HLA class I molecules in formalin-fixed paraffin-embedded tissue sections. However, we created a novel monoclonal pan-HLA class I antibody, EMR8-5, suitable for the immunostaining of formalin-fixed tissue specimens [13]. Therefore, we are now able to retrospectively investigate HLA class I expression levels in cancer specimens that were surgically resected and stored for a long time.

In this study, we investigated the prognostic value of HLA class I expression in patients with stage II colorectal cancer.

## Methods

### Patients

The study was approved by the Clinical Institutional Ethical Review Board of the Medical Institute of Bioregulation, Sapporo Medical University, Japan. We reviewed the clinical pathology archives of 97 consecutive patients with stage II (TNM classification [UICC]) colorectal cancer (61 men and 36 women; age range: 31–83 years) who underwent curative operation, defined as the removal of all of the tumoral masses, the absence of microscopic residual tumors, histology-negative resection margins, and lymphadenectomy extended beyond the involved nodes at the postoperative pathologic examination, at the Sapporo Medical University Hospital, Sapporo, Japan, from February 1994 to January 2005. Written informed consent was obtained from each patient according to the guidelines of the Declaration of Helsinki. Fifty-six patients with poorly differentiated histology or positive lymphovascular invasion had adjuvant chemotherapy. These patients were randomly assigned to receive 5-FU plus daily divided dose cisplatin (5-FU, 320 mg/m^2^ daily for 21 days; CDDP, 3.5 mg/m^2^ daily for 21 days) followed by oral 5-FU (200 mg/body daily for 2 years) or oral 5-FU therapy (200 mg/body daily for 2 years) exclusively as randomized trial [14]. No patients with rectal cancer had radiotherapy. Patients whose medical reports were incomplete were excluded. The median follow-up time was 54 months. Patients’ characteristics were assessed by tumor stage (stage IIA, stage IIB, and stage IIC), age, gender, tumor size, tumor location, histological type, and lymphovascular invasion.

### Antibody

The monoclonal anti-pan-HLA class I antibody EMR8-5 was established at our laboratory [13]. This mouse mAb (currently commercially available from Hokudo Co., Ltd., Japan) reacts with extracellular domains of HLA-A*2402, A*0101, A*1101, A*0201, A*0207, B*0702, B*0801, B*1501, B*3501, B*4001, B*4002, B*4006, B*4403, Cw*0102, Cw*0801, Cw*1202, and Cw*1502 [15] and shows strong reactivity in Western blots and conventional light microscopic analysis of formalin-fixed, paraffin-embedded sections.

### Immunohistochemistry

Immunohistochemical staining with the antibody was performed on formalin-fixed, paraffin-embedded tissues after steam heat-induced epitope retrieval. Subsequent incubations with a secondary biotinylated antibody, avidin-conjugated peroxidase complex, and chromogen were carried out on a Ventana NexES (Ventana Medical Systems, Inc., Tucson, AZ) [16]. Slides were then counterstained with hematoxylin, rinsed, dehydrated through graded alcohols into nonaqueous solution, and cover-slipped with mounting media. Positive reactivity to EMR8-5 was confirmed by staining of vascular endothelial cells and lymphocytes in sections of tumor specimens [15].

### Evaluation of HLA class I expression

**Figure 1:**
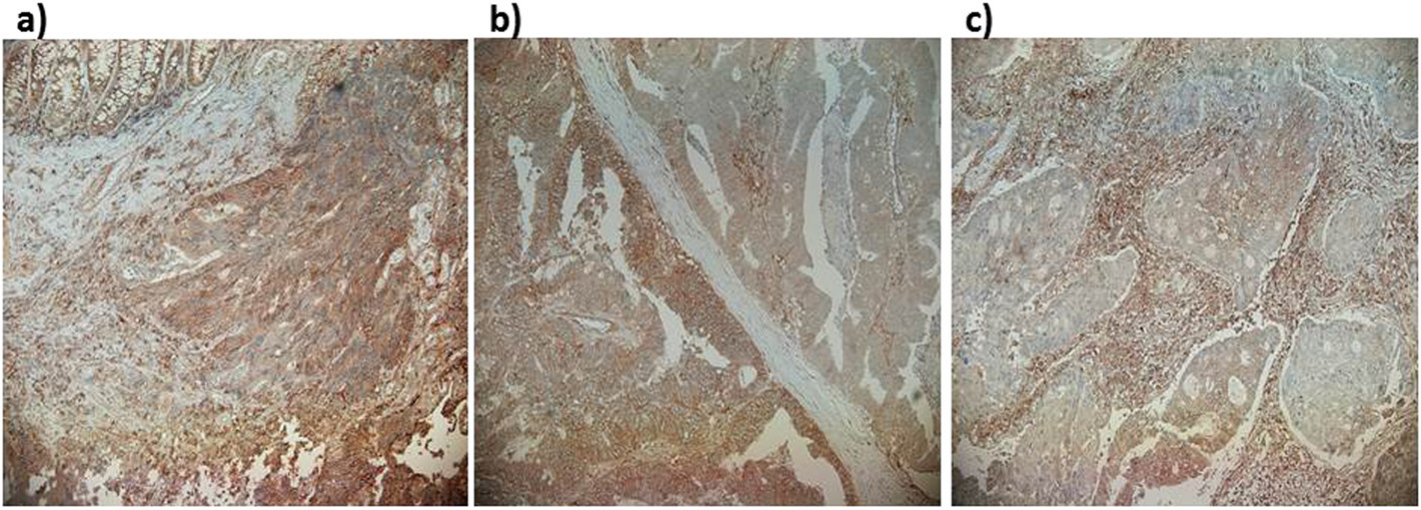
**Representative picture of immunostaining with the antibody EMR8-** **5.** The cancer cell membrane immunoreactivity level for HLA class I, which was expressed by EMR8-5, was classified into three categories (positive, dull, and negative). Positive was defined as complete and heterogeneous membrane staining in more than 80% of the tumor cells. Dull was defined as faint, incomplete, and heterogeneous membrane staining in 20% ~ 80% of the tumor cells. Negative was defined as membrane staining in less than 20% of the tumor cells. **(a)** Positive, **(b)** dull, and **(c)** negative.

The cancer cell membrane immunoreactivity level for HLA class I expressed by EMR8-5 was classified into three categories (positive, dull, and negative). Positive was defined as complete and heterogeneous membrane staining in more than 80% of the tumor cells (Figure 1a). Dull was defined as faint, incomplete, and heterogeneous membrane staining in 20% ~ 80% of the tumor cells (Figure 1b). Negative was defined as membrane staining in less than 20% of the tumor cells (Figure 1c). All specimens were reviewed independently using light microscopy in at least five areas at × 200 magnification by two investigators who were blinded to the clinicopathological data (TT and YI).

### Statistical analysis

We investigated the relationships between HLA class I expression levels and the other parameters (age, gender, tumor location, tumor size, depth, histological type, lymphovascular invasion, budding, number of lymph nodes analyzed after surgery (<12), HLA class I expression level, and adjuvant chemotherapy) and clinical outcome (disease-free survival: DFS). Some of these parameters (depth, histological type, lymphovascular invasion, budding, number of lymph nodes analyzed after surgery (<12)) were recommended as potential prognostic factors for curatively resected colorectal cancer by ESMO guidelines [8] or NCCN Guidelines Version 2 (2014). Statistical analysis was performed using SPSS Statistics 17.0. Deviation between the HLA class I expression level and clinicopathological parameters was evaluated with the Pearson *χ*^2^ test. Survival analysis was assessed by the Kaplan-Meier method, and the differences between survival curves were analyzed using the log-rank test. To evaluate the correlations between the survival rate and clinicopathological parameters, univariate and multivariate regression analyses according to the Cox proportional hazards regression model were used. A *P* value <0.05 was considered to indicate statistical significance.

## Results

### HLA class I expression level and patient characteristics in patients with stage II colorectal cancer

Immunohistochemical study of HLA class I in cancer cells revealed the following. There were 51 cases (53%) that were positive, which was defined as complete and heterogeneous membrane staining in more than 80% of the tumor cells, as well as 40 (41%) that were dull, which was defined as faint, incomplete, and heterogeneous membrane staining in 20% ~ 80% of the tumor cells, and six (6%) that were negative, which was defined as membrane staining in less than 20% of the tumor cells. In this study, the cases were divided into two groups, those that were “positive” (*n* = 51) and those that were “dull and negative” (*n* = 46). The relationships between HLA class I expression level and patients’ characteristics, i.e., tumor stage (stage IIA, stage IIB, and stage IIC), age, gender, tumor size, tumor location, histological type, and lymphovascular invasion, were assessed. The HLA class I expression level had no significant correlation with other clinicopathological parameters, except for gender (Table 1).

**Table 1:** **HLA class I expression levels and characteristics of the patients (stage II colorectal cancer)**

	**Positive**	**Dull and negative**	**Total**	***P*** **value**
	**(** ***n*** **= 51; 53%)**	**(** ***n*** **= 46; 47%)**	**(** ***n*** **= 97)**	
Stage				0.54
Stage IIA	46 (90)	42 (91)	88	
Stage IIB	2 (4)	0 (0)	2	
Stage IIC	3 (6)	4 (9)	7	
Age (years)				0.11
Mean ± SD	64 ± 9.7	60 ± 12.3		
Range	42 ~ 80	31 ~ 83		
Gender—no. of patients (%)				0.03
Male	27 (53%)	34 (74%)	61	
Female	24 (47%)	12 (26%)	36	
Diameter of primary tumor (mm)—no. (%)				0.87
≦30	11 (22%)	12 (26%)	23	
31–50	21 (41%)	17 (37%)	38	
≧51	19 (37%)	17 (37%)	36	
Location—no. of patients (%)				0.84
Right	16 (31%)	13 (28%)	29	
Left	15 (30%)	16 (35%)	31	
Rectum	20 (39%)	17 (37%)	37	
Histological type—no. (%)				0.23
Well/mod	48 (94%)	40 (87%)	88	
Por/muc	3 (6%)	6 (13%)	9	
Lymphatic invasion—no. of patients (%)				0.55
Negative	45 (88%)	40 (87%)	85	
Positive	6 (12%)	6 (13%)	12	
Venous invasion—no. of patients (%)				0.33
Negative	44 (86%)	42 (91%)	86	
Positive	7 (14%)	4 (9%)	11	

### Prognostic factors in patients with stage II colorectal cancer

Univariate analysis related to DFS revealed that the tumor location (*P* = 0.01) and HLA class I expression level (*P* = 0.02) might be significant prognostic factors among age, gender, tumor location, tumor size, depth, histological type, lymphovascular invasion, budding, number of lymph nodes analyzed, HLA class I expression level, and adjuvant chemotherapy. It also suggested that venous invasion might be a prognostic factor (*P* = 0.05). Moreover, multivariate analysis revealed that tumor location, HLA expression level, and venous invasion were significant independent prognostic factors (*P* < 0.05) (Table 2).

**Table 2:** **Univariate and multivariate analyses related to disease**-**free survival in 97 colorectal cancer patients**

**Variables**	**Univariate**		**Multivariate**	
	**Hazard ratio**	***P*** **value**	**Hazard ratio**	***P*** **value**
Age	0.98 (0.94–1.02)	0.38		
Gender (F)	1.42 (0.50–4.04)	0.51		
Tumor location (colon vs rectum)	4.23 (1.49–12.01)	0.01	4.11 (1.42–11.91)	0.009
Tumor size (≦5 cm)	0.64 (0.24–1.73)	0.38		
Tumor invasion (SI)	0.52 (0.12–2.28)	0.39		
Differentiation (por or muc)	1.50 (0.20–11.35)	0.70		
Lymphatic invasion (ly0, 1 vs ly2, 3)	1.10 (0.25–4.83)	0.90		
Venous invasion (v0, 1 vs v2, 3)	3.10 (1.00–9.56)	0.05	3.85 (1.15–12.92)	0.03
Budding	0.52 (0.19–1.41)	0.20		
Number of lymph nodes analyzed (<12)	1.32 (0.51–3.43)	0.57		
HLA expression level (dull or negative)	3.86 (1.26–11.85)	0.02	5.36 (1.68–17.11)	0.005
Adjuvant chemotherapy (no)	0.82 (0.30–2.22)	0.70		

### HLA class I expression and 5-year DFS

**Figure 2:**
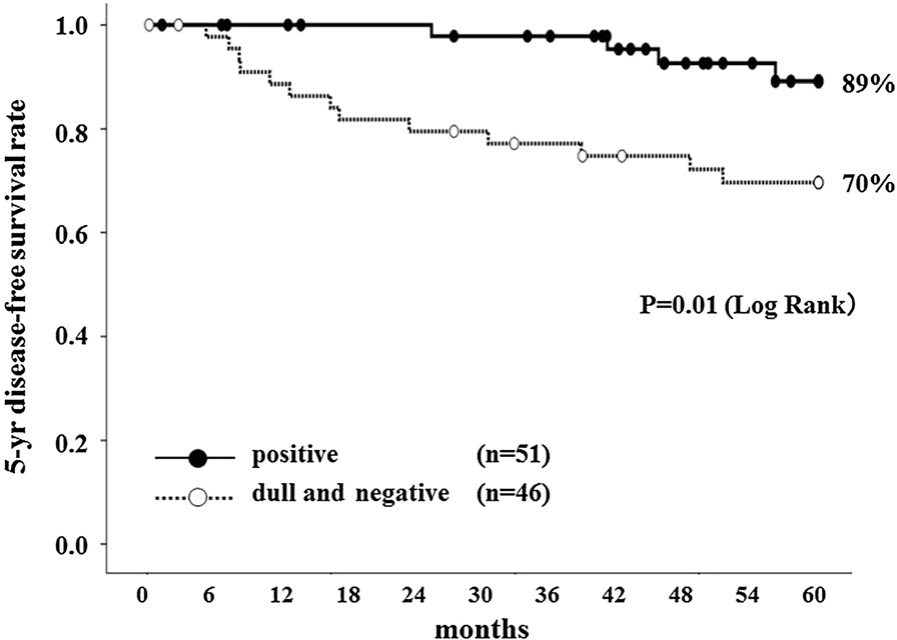
**Five-** **year disease-** **free survival curves of stage II colorectal cancer patients.** The 5-year DFS rates in the HLA class I positive group (*black circle*) and in the dull and negative group (*white circle*) were 89% and 70%, respectively. Patients with HLA class I positive expression had a significantly higher DFS rate than that of those with HLA class I dull and negative expression (*P* = 0.01).

Univariate and multivariate analyses revealed that the HLA class I expression level might be a useful prognostic factor related to DFS. Therefore, survival analysis was conducted using the Kaplan-Meier method. The 5-year DFS rates in the HLA class I positive group and in the dull and negative (dull/negative) group were 89% and 70%, respectively (*P* = 0.01) (Figure 2).

### HLA class I expression and adjuvant chemotherapy

**Figure 3:**
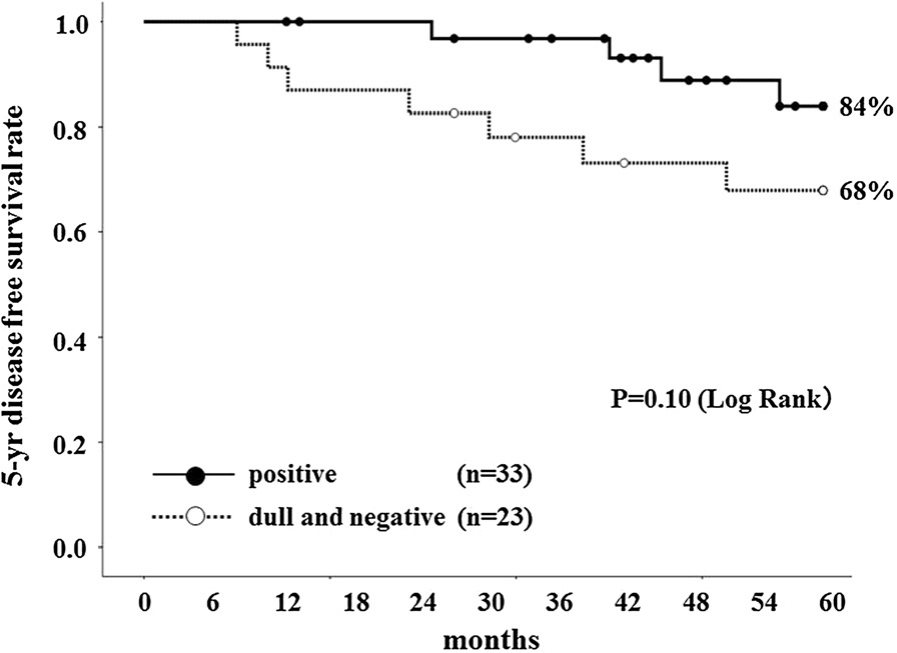
**Five-** **year disease-** **free survival curves of patients with adjuvant chemotherapy.** The 5-year DFS rates of patients with HLA class I positive expression (*black circle*) and with dull and negative expression (*white circle*) were compared. The 5-year DFS in patients with HLA dull and negative expression was decreased more than that of those with HLA positive expression, although there was no significant difference (*P* = 0.10).

**Figure 4:**
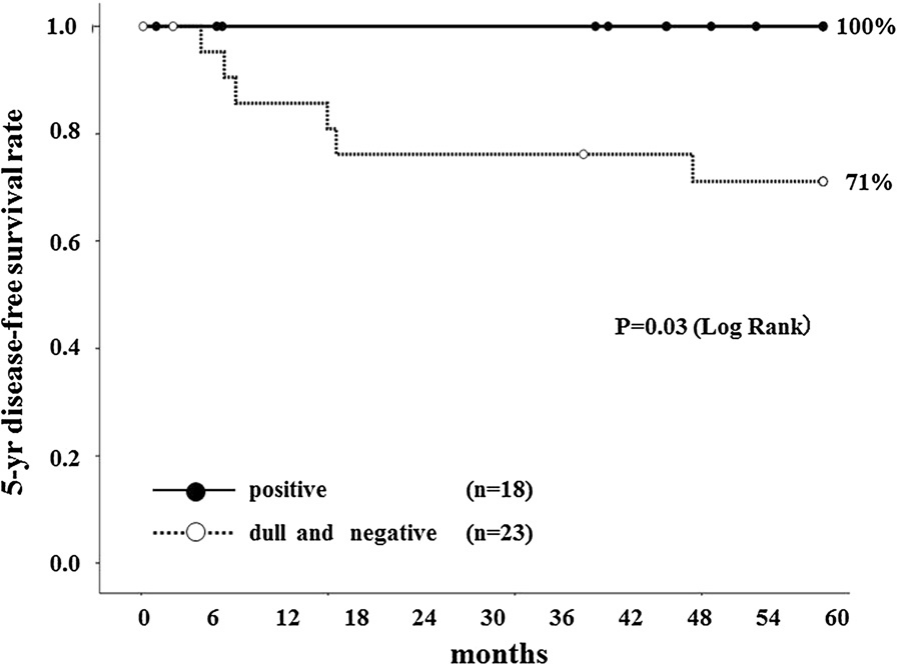
**Five-** **year disease-** **free survival curves of patients without adjuvant chemotherapy.** No patient with HLA class I positive expression (*black circle*) relapsed. Twenty-nine percent of patients with HLA dull and negative expression (*white circle*) relapsed. For patients without adjuvant chemotherapy, there was a significant difference in the 5-year DFS between patients with HLA class I positive expression and those with dull and negative expression (*P* = 0.03).

Fifty-six stage II colorectal cancer patients with poorly differentiated histology or positive lymphovascular invasion had adjuvant chemotherapy. For patients with this chemotherapy, the 5-year DFS rates of those with HLA class I positive expression and those with dull/negative expression were compared. The 5-year DFS rates in the HLA class I positive group and in the dull/negative group were 84% and 68%, respectively (Figure 3). The 5-year DFS in patients with HLA dull/negative expression was lower than that of those with HLA positive expression, although there was no significant difference (*P* = 0.10). On the other hand, no patient with HLA class I positive expression without chemotherapy relapsed, whereas 29% of those with HLA dull/negative expression relapsed. For those without adjuvant chemotherapy, there was a significant difference in 5-year DFS between patients with HLA class I positive expression and dull/negative expression (*P* = 0.03) (Figure 4).

## Discussion

Prognostic factors are useful for determination of the therapeutic strategy and follow-up examination after curative operation in cancer treatment. There are various reports of clinical and pathological prognostic factors. However, there are few immunological prognostic factors. The immunological state of the host can influence the prognosis for cancer patients as well as the features of the cancer.

HLA class I molecules have a central role in the anti-cancer immune system, especially as cancer antigen-presenting molecules for CTLs [13]. CTLs can recognize antigenic peptides presented on the cell surface by HLA class I molecules and kill target cells such as cancer cells. However, cancer cells can escape from the immune system by downregulation of HLA class I molecules, secretion of immunosuppressive cytokines, and infiltration of immunosuppressive cells [9-13]. One mechanism of recurrence after curative operation might be immune escape by micrometastatic cancer cells. Therefore, we focused on HLA class I molecules, key molecules in the immune system, to investigate the possibility of new immunological prognostic factors. This investigation was enabled through the use of the novel monoclonal pan-HLA class I antibody EMR8-5 [13], which is suitable for the immunostaining of surgically resected, formalin-fixed tissue specimens stored for a long time.

In this study, we investigated the HLA class I expression level and the prognoses of stage II colorectal cancer patients who underwent curative operation. In patients with stage II cancer, there was a significant difference in 5-year DFS between HLA class I positive patients and dull/negative patients (*P* = 0.01). Patients with HLA class I positive expression had a higher 5-year overall survival (OS) rate than those with HLA class I dull/negative expression, although there was no significant difference (*P* = 0.29) (data not shown). In addition, univariate and multivariate analyses revealed that the HLA class I expression level might be a significant independent prognostic factor. These data suggested that the HLA class I expression level might be a useful prognostic factor, particularly as a predictive factor for relapse, in stage II colorectal cancer. The reason why there was no significant difference in OS for stage II colorectal cancer patients is speculated to be that the beneficial treatments after recurrence might have more influence on OS than the immunological state in the living body such as the HLA class I expression level.

We have also reported that the HLA class I expression level might be a prognostic factor for other cancers such as osteosarcoma, clear cell renal cell carcinoma, and bladder cancer [15-19]. Tsukahara et al. reported that patients with osteosarcoma highly expressing HLA class I had significantly better OS and DFS than those with HLA class I-negative osteosarcoma [15]. Thus, there might be a difference in the impact of the HLA class I expression level on OS or DFS depending on the cancer. Although most reports, including our study, suggested that downregulation of HLA class I expression level was associated with a poor prognosis, Madjd Z et al. reported that total loss of HLA class I was an independent indicator of good prognosis in breast cancer [20]. They considered that the loss of HLA class I might make the tumors more susceptible to natural killer (NK) killing and result in a better prognostic outcome. It is due to the presence of HLA class I allele-specific killer cell inhibitory receptors (KIRs) on the surface of NK cells. Thus, in the absence of HLA class I expression, this KIRs-mediated inhibitory signaling is lost, resulting in the activation of NK cytolytic effector functions [21]. NK cell-mediated cytotoxicity is regulated by a delicate balance between activating and inhibitory signals. So, the prognostic influence brought by the HLA class I expression level might depend on the various cancer immune circumstances.

Surgery alone has relatively favorable results in colorectal cancer patients with stage II disease; hence, any advantage conferred by adjuvant chemotherapy after the curative operation is likely to be small. However, in real life in Japan, approximately 13% of patients with stage II colorectal cancer are found to have recurrence. The seventh edition of the American Joint Committee on Cancer (AJCC) Staging Manual divides stage II into three groups: stage IIA (T3N0), stage IIB (T4aN0), and stage IIC (T4bN0). There is a report that the prognoses for the stage IIB and IIC subgroups are worse than those of some stage III patients [22]. Therefore, stage II patients could be divided into high- and low-risk populations. We should select high-risk stage II patients and give adjuvant chemotherapy to prevent recurrence by micrometastases only to those patients who can obtain a significant benefit from it. The NCCN Guidelines Version 2 (2014) recommended the following risk factors for recurrence: number of lymph nodes analyzed after surgery (<12), poorly differentiated histology, lymphatic/vascular invasion, bowel obstruction, perineural invasion, localized perforation, and close, indeterminate, or positive margins. The ESMO consensus guideline recommended the following factors: lymph node sampling <12, poorly differentiated tumor, vascular or lymphatic or perineural invasion, T4 stage, and clinical presentation with intestinal occlusion or perforation [8]. In this study, patients with poorly differentiated tumors or moderate and severe lymphovascular invasion were considered to be high-risk stage II patients and underwent adjuvant chemotherapy. We investigated the 5-year DFS in stage II patients with and without adjuvant chemotherapy, respectively. Patients with HLA class I positive expression had a higher DFS rate than those with HLA class I dull/negative expression under both settings. In addition, for low-risk patients without chemotherapy, all patients with HLA class I positive expression did not relapse, although 29% of those with HLA class I dull/negative expression relapsed. These data might make certain of the prognostic value of HLA class I expression for relapse.

## Conclusions

The HLA class I expression level might be a very sensitive prognostic factor in colorectal cancer patients with stage II disease.

## Abbreviations

DFS: Disease-free survival
CEA: Carcinoembryonic antigen
CTLs: Cytotoxic T lymphocytes
ESMO: European Society for Medical Oncology
NCCN: National Comprehensive Cancer Network
NK: Natural killer
KIRs: Killer cell inhibitory receptors
AJCC: American Joint Committee on Cancer
